# The significance of a concomitant clavicle fracture in flail chest patients: incidence, concomitant injuries, and outcome of 12,348 polytraumata from the TraumaRegister DGU^®^

**DOI:** 10.1007/s00068-021-01819-5

**Published:** 2021-11-05

**Authors:** Mustafa Sinan Bakir, Andreas Langenbach, Melina Pinther, Rolf Lefering, Sebastian Krinner, Marco Grosso, Axel Ekkernkamp, Stefan Schulz-Drost, the TraumaRegister DGU

**Affiliations:** 1 Department of Trauma and Orthopedic Surgery, University Hospital Erlangen, Krankenhausstr. 12, 91054 Erlangen, Germany; 2 Department of Orthopaedics and Trauma Surgery, Klinikum Forchheim, Krankenhausstraße 10, 91301 Forchheim, Germany; 3 Department of Trauma and Reconstructive Surgery and Rehabilitative Medicine, Medical University Greifswald, Ferdinand-Sauerbruch-Straße, 17475 Greifswald, Germany; 4 Department for Trauma Surgery and Orthopaedics, BG Klinikum Unfallkrankenhaus Berlin gGmbH, Warener Str. 7, 12683 Berlin, Germany; 5 Department of Medicine, Institute for Research in Operative Medicine (IFOM), Faculty of Health, Universität Witten-Herdecke, Ostmerheimer Straße 200, 51109 Cologne, Germany; 6 Department for Trauma Surgery, Helios Hospital Schwerin, Wismarsche Strasse 393-397, 19049 Schwerin, Germany; 7 Committee on Emergency Medicine, Intensive Care, Trauma Management (Sektion NIS) of the German Trauma Society (DGU), Berlin, Germany

**Keywords:** Flail chest, Clavicle fracture, Costoclavicular injury, Trauma registry

## Abstract

**Purpose:**

Isolated clavicle fractures (CF) rarely show complications, but their influence in the thorax trauma of the seriously injured still remains unclear. Some authors associate CF with a higher degree of chest injuries; therefore, the clavicle is meant to be a gatekeeper of the thorax.

**Methods:**

A retrospective analysis of the TraumaRegister DGU^®^ (project 2017-10) was carried out involving the years 2009–2016 (ISS ≥ 16, primary admission to a trauma center). Cohort formation: unilateral and bilateral flail chest injuries (FC), respectively, with and without a concomitant CF.

**Results:**

73,141 patients (26.5% female) met the inclusion criteria and 12,348 had flail chest injuries (FC; 20.0% CF; 67.7% monolateral FC), 25,425 other rib fractures (17.7% CF), and 35,368 had no rib fractures (6.5% CF). On average, monolateral FC patients were 56.0 ± 17.9 years old and bilateral FC patients were 57.7 ± 19 years old. The ISS in unilateral and bilateral FC were 29.1 ± 11.7 and 42.2 ± 12.9 points, respectively. FC with a CF occurred more frequently with bicycle and motorbike injuries in monolateral FC and pedestrians in bilateral FC injuries and less frequently due to falls. Patients with a CF in addition to a FC had longer hospital and ICU stays, underwent artificially respiration for longer periods, and died less often than patients without a CF. The effects were highly significant in bilateral FC. CF indicates more relevant concomitant injuries of the lung, scapula, and spinal column. Moreover, CF was associated with more injuries of the extremities in monolateral CF.

**Conclusion:**

Due to the relevance of a concomitant CF fracture in FC, diagnostics should focus on finding CFs or rule them out. Combined costoclavicular injuries are associated with a significantly higher degree of thoracic injuries and longer hospital stays.

## Background

Upper quadrant injuries of the human body often affect the clavicle and ribs. Injuries to both of these areas can be attributed to isolated occurrences in the majority of patients who have rather complication-free courses and are consequently treated conservatively in most cases [1, 2]. Interestingly, however, the relevant association of a clavicle fracture (CF) in polytrauma with more frequent and complex additional injuries to the arm and thorax underlies the function of the clavicle as an important link between the functionally important arm and the thoracic trunk with its vital organ systems [1–4].

**Fig. 1:**
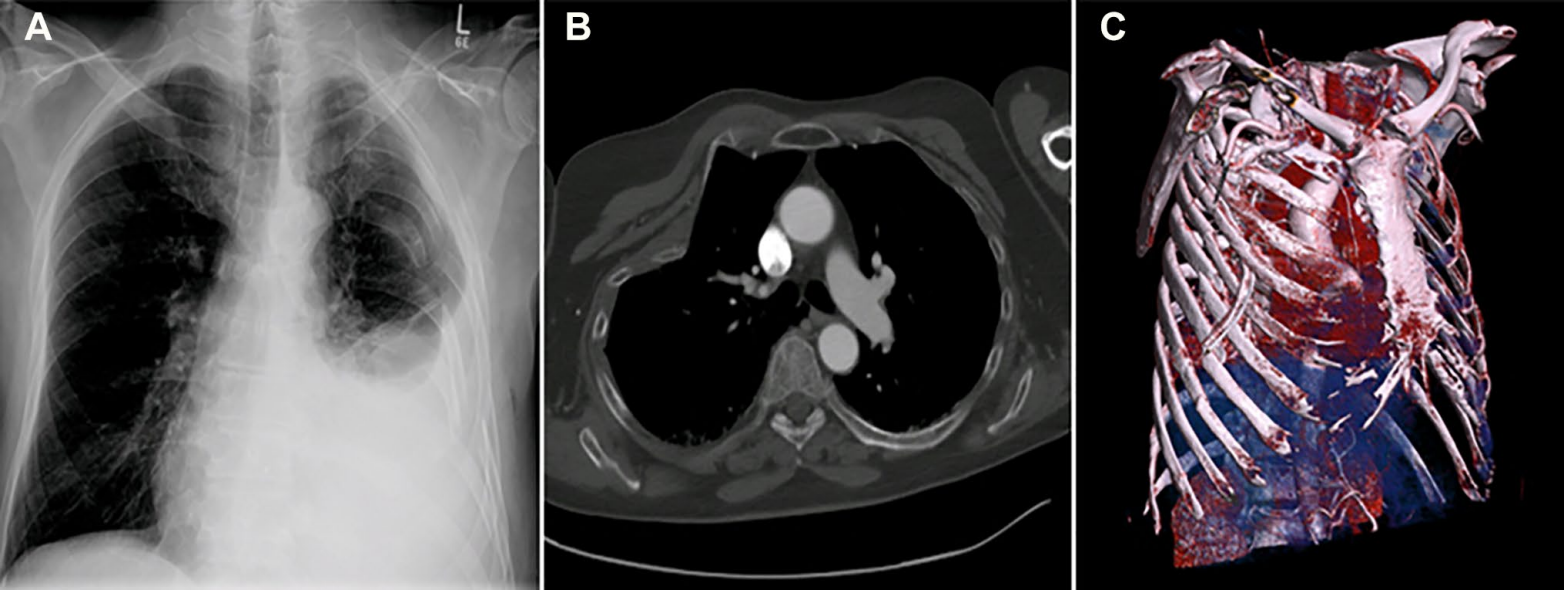
Clinical examples of costoclavicular injuries. **A** Left sided flail chest, lung restriction, pleural effusion. **B** Right sided severe deformity. **C** Right sided deformity, chest tube in hematothorax

As one of the most severe entities of thoracic trauma, the flail chest (FC) is often associated with significant complications [5]. FC is defined as at least three consecutive ribs broken in at least two places resulting in instability due to independently moving segments of the chest wall [5, 6]. More recently, however, the question of the importance of combined injuries to the thoracic wall and clavicle has become increasingly prominent. Some authors describe acutely occurring and secondary residual misalignments of the upper thoracic quadrant in costoclavicular injuries (CCI) as shown in Fig. 1 [7]. For this reason, the clavicle is considered the gatekeeper of the thorax [8].

This interesting finding will now be further examined in the context of a multi-center data collection of severely injured persons, mostly consisting of those from our nation and other participating countries in Europe. The present study aimed to test the primary hypothesis that an additional fracture of the clavicle in flail chest is more often associated with thoracic organ injuries and thus leads to a prolonged hospital stay.

## Methods

A retrospective analysis was carried out using the data from the TraumaRegister DGU^®^ (TR-DGU) of the German Trauma Society (DGU), which was founded in 1993. The purpose of this multicenter database is to provide a pseudonymized and standardized documentation of severely injured persons.

The data are collected prospectively in four consecutive phases: A—preclinical phase, B—emergency department (ED) and subsequent operation room phase, C—intensive care unit (ICU), and D—discharge. The documentation includes detailed information on demographics, injury patterns, comorbidities, preclinical and clinical management, intensive care history, important laboratory findings including transfusion data, and the outcome. The inclusion criteria are admission to the hospital via the ED, followed by intensive or intermediate care unit monitoring, or arrival at the clinic with vital signs and dying before admission to the ICU.

The scientific leadership is provided by the Committee on Emergency Medicine, Intensive Care and Trauma Management (Sektion NIS) of the German Trauma Society. Scientific evaluations are approved according to a review procedure established by Sektion NIS. The participating clinics are primarily located in Germany (90%), but an increasing number of clinics from other countries also contribute data. Almost 30,000 cases from over 650 hospitals are currently added to the database every year.

The present study is in line with the publication guidelines of the TraumaRegister DGU^®^ and registered as TR-DGU project ID 2017-010. All patients, their parents, or a legal guardian gave their informed written consent for collecting and publishing data. All data were collected anonymously and the study has been performed in accordance with the ethical standards laid down in the 1964 Declaration of Helsinki and its later amendments. Due to the retrospective character of the analysis, the given informed consent, the existing ethics vote, and the international character of the registry, no additional approval from local ethics committee was necessary (University Hospital Erlangen, Ethics Committee).

Specifically, patient data from 2009 to 2016 that involved an injury severity score (ISS) of ≥ 16 and a primary admission to a trauma center were included. Exclusion criteria consisted of several parameters to get the most homogeneous cohort possible: (1) age < 16 years (due to immature skeletal growth in children), (2) transfer from other hospitals (since no prior data from preclinical and first ED phase would be available), (3) early transfer to another hospital within a period of less than 48 h (since no final outcome would be available), (4) isolated brain injuries, and (5) cases treated in non-European hospitals.

Among the included patients, those suffering from rib fractures and those with unstable rib fractures corresponding to FC injury were identified. Monolateral and bilateral FC injuries were separated into subgroups, and the subsets with and without CF were examined for the following outcome parameters in these groups. According to our hypothesis that the CCI is a particularly severe injury entity, and our research question whether CCI should accordingly be treated with special focus, we take concomitant thoracic organ injuries and the length of hospital stay as primary outcome parameters. Secondary endpoints include preclinical and clinical outcome parameters as age, sex, ISS, head injury, trauma mechanism (blunt/penetrating), shock status at admission, year of trauma, and level of receiving hospital. Statistical analysis was performed using SPSS (Version 22 IBM, Armonk, USA). The tests used for the analysis were Fisher’s exact test for percentages and the Mann–Whitney *U* test for measures. The significance level was set at *p* < 0.01. A multivariate logistic regression analysis was performed with hospital mortality as the dependent variable.

## Results

**Fig. 2:**
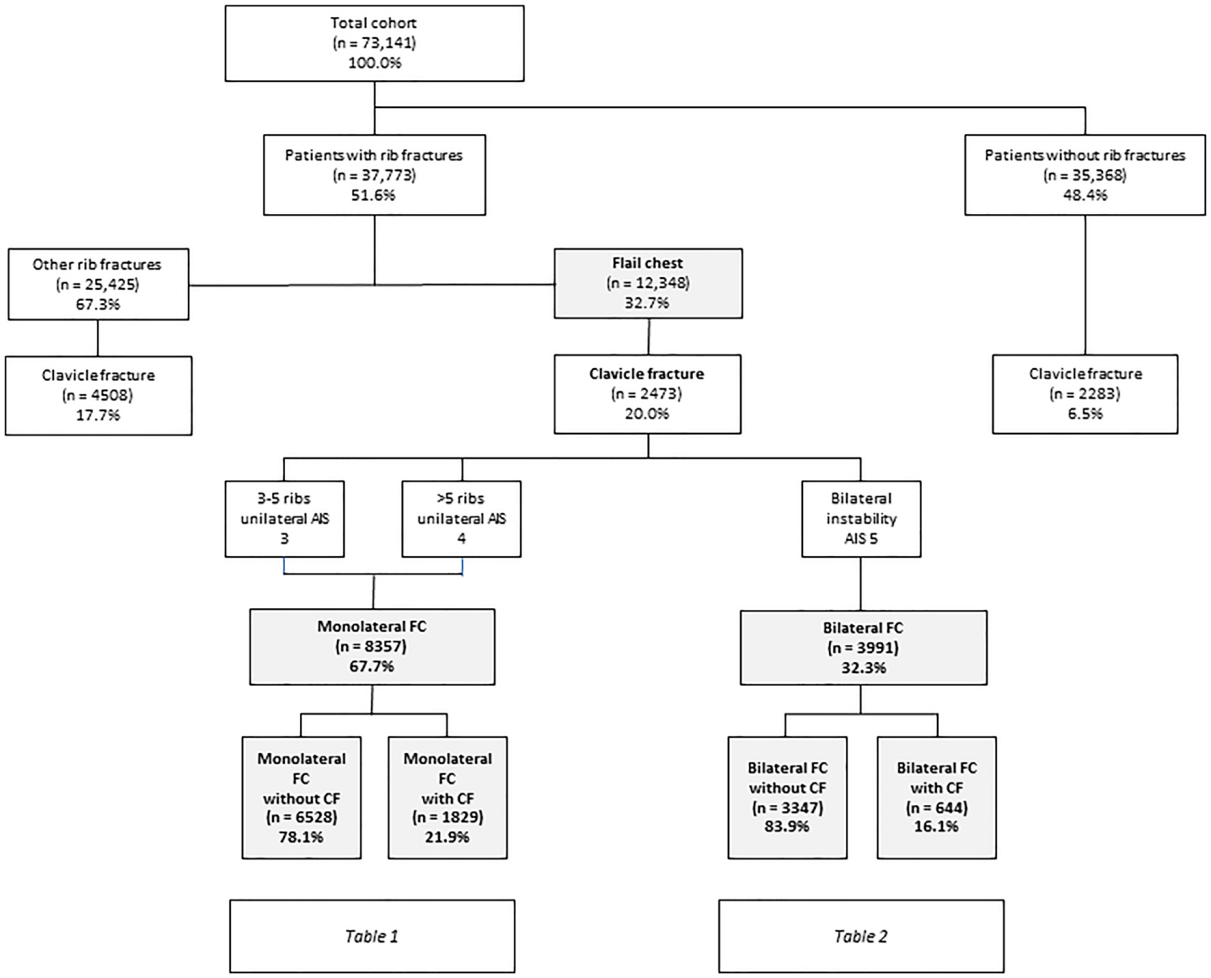
The collective. Distribution of the respective injury entities of costoclavicular injuries (*n* = number of patients, *FC* flail chest, *CF* clavicle fracture)

Overall 73,141 patients were included, 12,348 of which (32.7%) suffered from unstable serial rib fractures (Fig. 2). Finally, 8357 patients had monolateral flail-chest injuries (21.9% of these had clavicular fractures), and 3991 had bilateral flail-chest injuries (16.1% of these had clavicular fractures) as shown in Fig. 2.

### Patient characteristics

On average, the patients with flail-chest injuries (both mono and bilateral) were in their late 50’s (Tables 1 and 2). The mean ISS was 29.1 (± 11.7) for unilateral and 42.2 (± 12.9) for bilateral flail-chest injuries. Neither age nor overall injury severity was dependent on the presence of a clavicle fracture (Tables 1 and 2).

**Table 1:** Characteristica of monolateral flail chest

Monolateral flail chest AIS 3: n = 3228 AIS 4: n = 5129		Flail chest (AIS 3 + 4) *n* = 8357	Without CF *n* = 6528	With CF *n* = 1829	*p* value
Demography					
Age	Mean (SD)	56.0 (17.9)	56.0 (18.2)	55.7 (16.7)	0.28
Sex (Male)	%	76.3	75.7	78.2	0.029
Trauma					
Penetrating Trauma	%	1.5	1.7	1.0	0.060
ISS	Mean (SD)	29.1 (11.7)	29.1 (12.0)	29.2 (10.9)	0.023
Prehospital parameters					
GCS ≤ 8 at scene	%	20.6	20.7	20.6	0.95
Intubation	%	34.7	35.1	33.3	0.16
Chest tube	%	10.3	10.5	9.9	0.60
Shock (systolic BP ≤ 90)	%	16.4	17.5	12.8	< 0.001
In-hospital parameters					
Shock at admission	%	15.9	17.0	12.1	< 0.001
Chest tube in ED	%	39.5	38.8	41.8	0.086
Intensive care	%	91.3	90.7	93.5	< 0.001
Length of stay on ICU (days)	Mean Median (SD)	10.5 5 (13.4)	10.3 5 (13.8)	10.9 6 (11.9)	< 0.001
Mechanical ventilation (days)	Mean Median (SD)	5.7 1 (10.5)	5.6 1 (10.8)	5.8 1 (9.5)	0.11
Sepsis	%	11.5	12.0	9.7	0.057
Multi organ failure	%	34.0	34.5	32.2	0.22
Stay in hospital	Mean Median (SD)	21.7 17 (19.4)	21.7 17 (20.1)	21.8 18 (17.1)	< 0.001
Operative treatment of the ribs	%	19.1	18.5	21.4	0.039
Concomitant injuries					
Head AIS ≥ 3	%	30.3	28.9	35.2	< 0.001
Cervical spine AIS ≥ 2	%	11.6	11.3	13.0	0.043
Thoracic spine AIS ≥ 2	%	21.9	21.8	22.3	0.70
Lumbar spine AIS ≥ 2	%	21.0	22.2	16.7	< 0.001
Abdomen AIS ≥ 2	%	28.2	30.3	20.7	< 0.001
Pelvis AIS ≥ 2	%	25.7	27.5	19.0	< 0.001
Lower extremities AIS ≥ 2	%	25.2	27.2	18.4	< 0.001
Humerus	%	7.9	8.6	5.5	< 0.001
Scapula	%	19.0	15.7	30.6	< 0.001
Chest AIS ≥ 2					
Pneumothorax	%	54.8	53.0	61.1	< 0.001
Hemothorax	%	35.5	35.1	37.1	0.12
Lung contusion	%	47.0	45.9	50.8	< 0.001
Lung parenchyma	%	4.0	3.8	4.9	0.031
Heart injury AIS ≥ 1	%	4.8	5.0	4.0	0.072
Aorta	%	1.4	1.5	1.0	0.21
Great vessels AIS ≥ 2	%	2.2	2.3	2.2	0.93
A. subclavia	%	0.3	0.3	0.6	0.028
A. carotis	%	0.4	0.4	0.3	0.52
Brachial plexus AIS ≥ 2	%	0.3	0.1	0.7	< 0.001
Clavicle joints	%	2.0	2.3	1.0	< 0.001
Died within 24 h	%	8.6	9.5	5.2	< 0.001
Died during hospital stay	%	15.9	17.1	11.9	< 0.001
Outcome Scale (survivor only)					
2 = PVS	%	1.7	1.7	1.5	0.67
3 = major handicap	%	9.9	9.8	10.4	
4 = minor handicap	%	28.8	29.1	28.0	
5 = well recovered	%	59.5	59.3	60.2	

*AIS* Abbreviated Injury Scale, *n* = number of patients, *CF* clavicle fracture, *SD* standard deviation, *ISS* Injury Severity Score, *GCS* Glasgow Coma Scale, *BP* blood pressure, *ED* emergency department, *PVS* persistent vegetative state

**Table 2:** Characteristics of bilateral flail chest

Bilateral flail chest AIS 5: *n* = 3991		Flail chest (AIS 5) *n* = 3991	Without CF *n* = 3347	With CF *n* = 644	*p* value
Demography					
Age	Mean (SD)	57.7 (18.0)	58.1 (18.0)	55.7 (18.1)	0.003
Sex (Male)	%	72.2	72.3	72.2	0.75
Trauma					
Penetrating trauma	%	1.6	1.7	1.3	0.60
ISS	Mean (SD)	42.2 (12.9)	42.3 (13.1)	42.0 (11.5)	0.82
Prehospital parameters					
GCS ≤ 8 at scene	%	36.2	37.1	31.5	0.008
Intubation	%	53.5	53.9	51.4	0.26
Chest tube	%	16.3	16.6	15.1	0.54
Shock (systolic BP ≤ 90)	%	31.2	32.2	25.8	0.003
In-hospital parameters					
Shock at admission	%	32.0	32.5	29.4	0.15
Chest tube in ED	%	46.7	45.2	54.5	0.001
Intensive care	%	83.4	81.7	92.4	< 0.001
Length of stay on ICU (days)	Mean Median (SD)	12.5 7 (15.7)	12.0 6 (15.5)	15.6 11 (16.2)	< 0.001
Mechanical ventilation (days)	Mean Median (SD)	7.8 1 (12.3)	7.3 1 (12.0)	10.4 5 (13.5)	< 0.001
Sepsis	%	15.7	15.8	15.3	0.87
Multi-organ failure	%	50.3	50.2	50.7	0.91
Stay in hospital	Mean Median (SD)	22.6 18 (24.4)	21.9 16 (24.7)	26.5 23 (22.5)	< 0.001
Operative treatment of the ribs	%	21.4	21.3	22.0	0.40
Concomitant injuries					
Head AIS ≥ 3	%	35.3	34.9	37.3	0.24
Cervical spine AIS ≥ 2	%	16.7	15.9	21.1	0.002
Thoracic spine AIS ≥ 2	%	31.9	31.0	36.5	0.006
Lumbar spine AIS ≥ 2	%	25.2	25.0	26.2	0.52
Abdomen AIS ≥ 2	%	31.4	31.2	32.9	0.38
Pelvis AIS ≥ 2	%	32.6	32.7	32.0	0.71
Lower extremities AIS ≥ 2	%	33.3	33.5	32.3	0.55
Humerus	%	9.7	9.6	10.6	0.47
Scapula	%	16.1	13.1	31.2	< 0.001
Chest AIS ≥ 2					
Pneumothorax	%	52.9	50.5	65.4	< 0.001
Hemothorax	%	37.1	35.6	45.2	< 0.001
Lung contusion	%	42.8	40.4	55.1	< 0.001
Lung parenchyma	%	4.0	4.1	3.9	0.91
Heart injury AIS ≥ 1	%	7.5	7.1	9.3	0.060
Aorta	%	2.8	2.9	2.2	0.36
Great vessels AIS ≥ 2	%	4.4	4.7	2.8	0.035
A. subclavia	%	0.4	0.4	0	0.15
A. carotis	%	0.8	0.6	2.0	0.001
Brachial plexus AIS ≥ 2	%	0.2	0.1	0.6	0.043
Clavicle joints	%	1.2	1.0	2.0	0.047
Outcome					
Died within 24 h	%	23.6	25.5	13.8	< 0.001
Died during hospital stay	%	34.3	36.4	23.4	< 0.001
Outcome Scale (survivor only)					
2 = PVS	%	2.8	2.8	2.5	0.91
3 = major handicap	%	15.5	15.3	16.5	
4 = minor handicap	%	36.1	36.2	35.4	
5 = well recovered	%	45.6	45.6	45.6	

AIS = Abbreviated Injury Scale; *n* = number of patients; CF = clavicle fracture; SD = standard deviation; ISS = Injury Severity Score; GCS = Glasgow Coma Scale; BP = blood pressure; ED = emergency department; PVS = persistent vegetative state

### Associated injuries

Both groups show a significantly higher incidence of concomitant scapular fractures and thoracic injuries, such as pneumothorax, hemothorax, and pulmonary contusion, in cases in which an additional CF was found. For rare lung parenchymal and heart injuries, and other injuries to large vessels, no relevant differences could be identified except for carotid artery injuries, which were more common in bilateral rather than unilateral CCI. Injuries of the brachial plexus, although rare, were more frequently observed with concomitant CF (Tables 1 and 2).

### Trauma mechanism

**Fig. 3:**
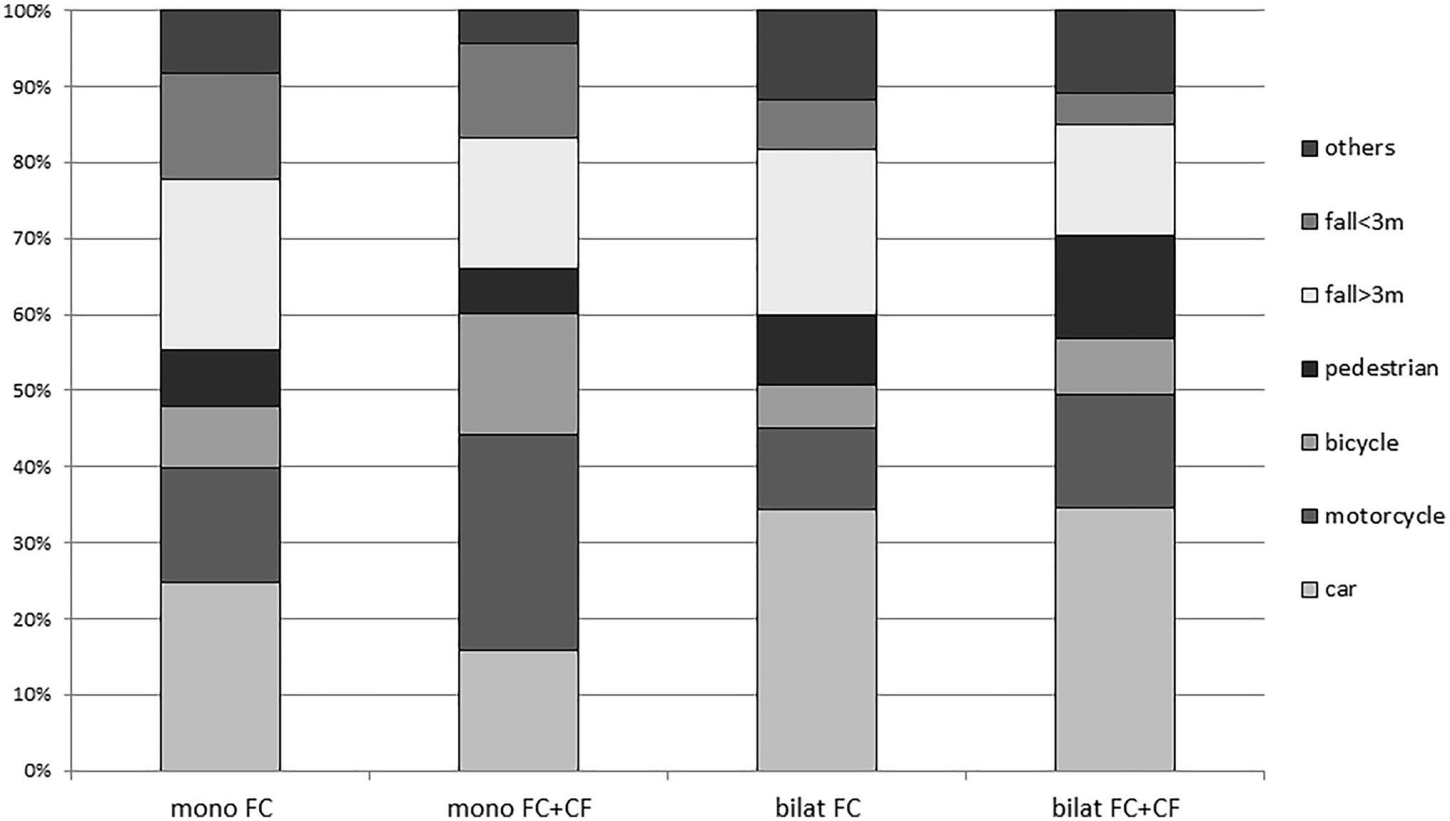
Trauma mechanism in the different groups. Both groups with a CF suffered significant more bicycle and motorbike accidents than the particular control group. Vice versa more falls occurred in the groups without CF (mono = monolateral, bilat = bilateral, FC = flail chest, CF = clavicle fracture)

While in both mono- and bilateral FC injuries without CF the trauma mechanisms of vehicle occupants and patients who underwent falls from significant heights dominated, those patients with additional CF, especially the monolateral injuries, were predominantly involved in motorbike and bicycle accidents as the cause of the injury (Fig. 3). Patients with bilateral FC injuries were more frequently found in pedestrians involved in an accident with a motor vehicle (Fig. 3).

### Preclinical parameters

No significant differences between the groups regarding the preclinical parameters of intubation and chest tube need were noted. The proportion of patients with circulatory shock was higher in both groups of FC injuries without CF (Tables 1 and 2).

### Clinical parameters

In both monolateral and bilateral FC, the proportion of patients undergoing intensive care was significantly higher when a CF had occurred. Furthermore, this group also showed a longer stay in the ICU, longer duration of intubation, and longer total hospital stay than those without a CF. In addition, chest tubes were more frequently applied in cases in which an additional CF had occurred. The rib fractures were treated more frequently by surgery in CCI, significantly in monoliteral CCI, than in FC without CF (Tables 1 and 2).

### Outcome

Patients died significantly more frequently in case of chest trauma without an additional CF. Multivariate logistic regression analysis showed a lower mortality rate in the group with CF in contrast to the group without CF (odds ratio [OR] = 0.62; 95% confidence interval [CI] 0.53–0.72), while the type of FC (unilateral/bilateral) did not have an effect on mortality (OR 0.97; 95% CI 0.82–1.14). In the case of survival, no relevant differences in the functional outcome subgroups were found (Tables 1 and 2).

## Discussion

The present study considers an international European collective of severely injured persons with chest trauma and the influence of an additional CF. While isolated CF were rarely observed at 6.5%, about one in six patients in the collective had suffered an unstable thoracic injury and one in five out of them an additional CF (Fig. 2). Overall, the costoclavicular combination injury in FC was rather rare at 3.4%.

Regarding the abbreviated injury score (AIS) and ISS of the overall injury severity, no relevant difference in CF could be identified [9]. This finding is also easy to understand since on one hand, a CF with an AIS of 2 is not considered a serious injury, but on the other hand, the high injury severity of the thorax in terms of serial rib fractures in monolateral injury is related to AIS 3 and 4 and even to AIS 5 injuries, which correspond to a life-threatening injury, in bilateral fractures.

These findings support the hypothesis that a CF is significantly and more frequently associated with thoracic organ injuries, such as pneumothorax, hematothorax, and pulmonary contusion, in the examination of this large collective, which is similar to the results of Horst et al. and van Laarhoven et al. [3, 4, 7]. Horst et al. showed that the CF is not only more frequently associated with additional thoracic and upper extremity injuries but also longer periods of intensive care and hospitalization as well as more complications were seen [1, 2]. In a large monocentric collective, van Laarhoven’s group amplified the importance of an additional CF in chest trauma, in particular, the finding that an additional CF was associated with a higher overall injury severity and with more concomitant thoracic injuries [8]. However, no significant relationship could be established for the rare lung parenchymal injuries when FC already occurred, whereas CF is known as a significant predictor for thoracic injuries in the whole collective what includes the lung injuries [3].

The higher rate of thoracic organ injuries is reflected in both groups of CCI as higher rates of patients in the ICU and higher rates of chest tubes, resulting in significantly longer hospital stays and intubation times. The effects of bilateral thoracic injuries were particularly pronounced. Although a statistically significant difference for monolateral FC was found accordingly, we do not consider it to be clinically significant concerning the length of stay in hospital/ICU or in terms of mechanical ventilation, since the maximum difference is both for median and mean of one day. The bilateral CCIs were significantly more severely affected in contrast to the monolateral CCI regarding in-hospital parameters and to a smaller extent, the same applies to FC injuries without CF. We took this finding as a sign of confirmation of our hypothesis that states that CCI is a separate and more severe injury entity. Interestingly, however, no relevant difference was seen in the occurrence of sepsis or multiple organ failure. The patients with additional CF apparently recovered well enough during their longer stays.

On the other hand, the mortality rate was higher if no CF had occurred, which agrees with the findings from a study by Horst et al. consisting a whole collective of polytraumatized patients [3]. In both groups, the number of patients with prehospital shock was significantly higher without a CF, which could be a potential explanation for increased mortality. In the case of monolateral FC injury, higher mortality and preclinical shock could be explained by a significantly higher injury rate of the abdomen and the pelvis, whereas no significant differences in the bilateral FC group were identified in this regard. Ultimately, no valid cause for the higher patient mortality without CF can be certainly derived from the available data due to the impossibility of tracing the data in a retrospective study design. However, FC on its own is associated with relevant mortality [10]. The results contradict those of the published monocentric collective from van Laarhoven et al. in which an additional CF was associated with higher mortality rates (21.9% versus 17.8% in patients without CF) [8]. Interestingly, a similar observation to that described in our study was shown for chest trauma with concomitant scapular fracture; the group with scapular fractures also showed lower mortality although significant more frequent thoracic organ injuries occurred [11]. So far, this association remains unclear for scapular and clavicular fractures.

However, a hypothetical explanation could be the different frequency of surgical treatment of the rib fractures and their possible associated advantages [12, 13]. Significantly more of these operations were performed in monolateral CCI than in monolateral FC without CF. Since the entity of the CCI as a combination injury may have given the indication for operative stabilization, the improved post-operative stability in the CCI group would explain a lower mortality through potentially reduced respiratory failure during its course [7]. Similarly, recent work has shown that surgical management of FC can be a promising and beneficial therapy strategy [2, 12–17]. However, some studies were rather cautious with regard to surgical intervention of rib fractures so that the literature remains somehow controversial [18–20]. Analogous to the advantages of surgical FC repair, our results encourage the authors to believe that the majority of patients with CCI benefit more from surgical than from conservative therapy [2, 7, 21].

In cases of bilateral FC, the presence of a CF seems to be of minor importance in the decision to undertake surgical therapy. This finding can be explained by the fact that in a highly unstable bilateral situation, the indication for surgery is more likely to be made and is independent of a CF, which otherwise represents a further instability factor in monolateral FC [7, 22].

Another hypothesis for the obviously contradictory relationship that a CF is associated with more thoracic (organ) injuries, but is contemporaneously a predictor of lower mortality, could be the missing causal relationship between mortality and prevalence of a CF. The mortality seems to be related to other confounders that influence and worsen the outcome, but are not included in the trauma registry used. These factors could be parameters, which might be related to the severity of the flail chest and resultant injuries, such as the rate of pneumonia/tracheostomy/respiratory failure or the RibScore [23]. These aspects should also be analyzed in a prospective study, which should be carried out to confirm/disprove the associations we have shown. Since the RibScore still contains additional morphological parameters such as information about (bicortical) displacement of rib fractures or their presence in all three anatomic areas (anterior, lateral, and posterior) or involvement of the first rib, it might be possible to prove our suggested relationships [23].

What was striking in the analysis was the significantly lower proportion of patients with bilateral FC without CF in the ICU compared to the group with bilateral CCI although both had very high ISS scores. Mortality was also significantly higher in this group. We assume that if only a small proportion was admitted to the ICU, this process could be biased toward higher mortality since monitoring and treatment of these high-risk patients cannot be ensured equivalently in a peripheral ward. However, it should also be noted in this context that this group of bilateral FC patients without CF was significantly older. Due to their increased age, these patients may already have a higher number of pre-existing comorbidities leading to therapy limitation. This finding might be a potential explanation for the apparent contradiction and, of course, increased age can also be a reason for higher mortality by itself.

### Trauma mechanism

On the other hand, the trauma mechanisms have been determined to have a comprehensive distribution (Fig. 3). It is well known that CF is often caused by accidents during sporting events and by traffic accidents in the context of multiple injured patients [24].

Our study supports these findings, for example, as in severe bilateral thoracic injury an association between additional CF and injuries in motorcyclists, cyclists, and pedestrians, was found, whereas in monolateral thoracic injury, an additional CF is highly significant and more common in motorbike and bicycle accidents. Although a causal association between the point of impact and the injury pattern cannot be made using our data set, this finding indicates that an association between the CF and a side impact on the upper trunk of the body exists [25]. This impact is also felt to be the main cause of a CF [26, 27]. In addition, monolateral unstable chest wall injuries are generally caused by a side impact [28]. In the cascade of the load effect, the clavicle will first break and only in the wake of another intrusion, for example, the underlying ribs. Above all, the upper ribs (second, third, and fourth ribs), in addition to the first rib are rarely affected. Since they are also generally associated with a higher risk of concomitant thoracic organ injuries, our results are consistent with this thesis [29, 30]. Similar to the results of van Laarhoven et al., the present collective also shows a significantly higher rate of organ injuries when the clavicle breaks in addition to rib breakage [8, 9].

### Functional considerations

While un-displaced CFs usually heal very well with conservative therapy, dislocated CFs may be associated with complications. In addition to delayed or even absent fracture healing, a permanent malposition can be accompanied by a cosmetically disturbing humpback formation and in the worst case, shortening and limitations of shoulder mobility. Therefore, an operative osteosynthesis should be considered in these cases [31–33].

If the attached components of the shoulder girdle, such as the scapula and ribs, are included in the functionality of the shoulder, the relevance of a preserved thoracoscapular plain bearing is quickly emphasized. However, this event is often disturbed in CCI by dislocated rib fractures in its congruence. As a late consequence, this process can result in a scapular snapping syndrome [34]. However, concomitant CF in these cases is largely responsible for the instability of the shoulder girdle. In a monocentric data collective, Stahl et al. were able to show that additional rib fractures can lead to an increased frequency of dislocation of clavicular shaft fractures [22]. Frequently, a lowering of the shoulder is observed in the CCI because, the entire upper quadrant of the trunk is unstable (Fig. 1). These resulting primary or secondary residual misalignments can lead to permanent, disfiguring deformities of the shoulder girdle and functional restrictions [7]. Therefore, surgical stabilization should also be considered for dislocated CF and rib fractures in CCI since spontaneous correction of the deformity cannot be expected [7, 21, 35]. Thus, the CF is a special case in the spectrum of all CFs when occurring in connection with a CCI, in spite of the conservative therapy that is often performed for a single CF, since it should clearly be treated surgically.

### Limitations

Given the retrospective character of this investigation, it is inherent in this study type that interpretation of the associations shown has to be done carefully. Since retrospective analyzes do not allow any conclusions to be drawn about a causal relationship, this must be mentioned as a main limitation. Therefore, our associations should be re-evaluated in a prospective study design to confirm or refute our conclusions.

Concerning mortality, a decrease in mortality in the CCI group could also lead to a longer hospital stay since the highest mortality is usually reported in the first days of the inpatient stay [36]. Therefore, a longer length of stay/length of mechanical ventilation would not necessarily indicate a more severe injury since a high mortality could be a bias. Another blind spot in our analysis occurred because no statement about the cause of mortality can be made. Therefore, no conclusion about a potential respiratory failure due to the FC is possible, which are known for an increased risk of a negative pulmonary outcome [37]. The causes of mortality were not recorded in this registry. A record of possible therapy limitations on the part of the patient’s/relative’s request or a palliative situation was also not made. In summary, the mortality results should, therefore, be interpreted in a differentiated way. Due to the inhomogeneous documentation of the surgical interventions in the registry over the years, it is not possible to make reliable statements about the frequency of surgically treated CFs in CCIs. Therefore, it is not possible to show an exact association whether the operation of a CF was more often performed in connection with surgical chest wall repair.

Due to the high number of cases, this study allows a suitable overview of the incidence of these injuries and their influence on the entire clinical process. Despite this stronger significance level, even minor differences could become statistically significant due to the large sample size. However, in the nature of a registry study with retrospective evaluation, case-related clinical details cannot be analysed, and causal conclusions cannot be drawn. Therefore, it is not possible to give an exact statement on the severity of individual (concomitant) injuries. In the case of pulmonary contusions, for example, the AIS classification used is applied for severity definition, but a more detailed scoring method cannot be used retrospectively [38, 39]. Due to the missing opportunity for double-checking, it is also important to rely on the accuracy of the person entering the data, as this determines the data precision and quality [40]. Moreover, the present study cannot include and present detailed morphological properties [21]. It would be particularly interesting to determine which ribs break and at which locus they break to define a dependency. Examining this process should be part of further detailed studies involving the morphological and fracture kinetic aspects as the relationship between trauma impact and injury pattern and the establishment of therapeutic strategies, particularly with regard to a common surgical approach of ribs and clavicle.

## Conclusions

The present study emphasizes the importance of additional clavicle fractures in chest trauma in an extremely meaningful international collective and reveals an association with an increase in the rate of thoracic organ injuries and the need for more extensive intensive care. The importance of flail chest injuries as the most severe chest injury was confirmed by our investigation, and the high value at AIS score could thus be emphasized, especially for bilateral FC injuries. Any CF should be identified or excluded during the initial care of the seriously injured, and the presence of a relevant thoracic organ injury should always be taken into account and vice versa, especially in cases of motorbike and bicycle accidents. Therefore, special focus should be placed on the combination of FC and CF as these CCIs seem to predict the maximal risk of respiratory failure due to the highest severity of a thoracic injury. Based on the expected advantages in the outcome, CCI should be considered for surgical treatment via stabilization of the chest wall and the clavicle together.

## Data Availability

The data that support the findings of this study are available from AUC—Academy for Trauma Surgery (AUC—Akademie der Unfallchirurgie GmbH), a company affiliated to the German Trauma Society, but restrictions apply to the availability of these data. The data were used under license for the current study according to the publication guideline of TraumaRegister DGU®, and so are not publicly available. Data are, however, available from the authors upon reasonable request and with permission of AUC—Academy for Trauma Surgery and the TraumaRegister DGU^®^ Review Board.
